# Negative association of C-reactive protein-albumin-lymphocyte index (CALLY index) with all-cause and cardiovascular mortality in population with CKD: the mediating role of biological age acceleration

**DOI:** 10.1080/0886022X.2025.2586892

**Published:** 2025-11-18

**Authors:** Dongli Huang, Hang Wu, Juan Zhou, Supei Yin, Ruiqiong Wang, Zhihui Quan

**Affiliations:** Bishan Hospital of Chongqing Medical University, Chongqing, China

**Keywords:** C-reactive protein-albumin-lymphocyte index, mortality, chronic kidney disease, NHANES, mediation analysis, biological aging

## Abstract

This study investigated the association between the C-reactive protein-albumin-lymphocyte (CALLY) index and all-cause mortality and cardiovascular disease (CVD) mortality in patients with chronic kidney disease(CKD) while exploring biological aging as a potential mediator. This study included 4,515 patients with CKD from the National Health and Nutrition Examination Survey (NHANES) conducted between 1999 and 2010. The CALLY index was assessed only at baseline. The association between the CALLY index and all-cause and cardiovascular mortality was analyzed using Cox proportional hazards models. Additionally, Kaplan–Meier curves, smooth curve fitting, segmented linear regression, and various subgroup and sensitivity analyses were performed. Finally, the mediating role of biological age acceleration was explored. Among 4,515 participants, with a median follow-up of 127 months and 2,264 recorded deaths, a higher Ln-CALLY was associated with a lower risk of all-cause mortality (HR = 0.845, 95% CI: 0.817, 0.874) and cardiovascular disease (CVD) mortality (HR = 0.860, 95% CI: 0.809, 0.915). Smoothed curve fitting and threshold effect analyses indicated that the effect was more pronounced when Ln-CALLY was lower than 4.96. Mediation analyses further revealed that biological age acceleration (BioAgeAccel) mediated 14.168% of the association between CALLY and all-cause mortality and 27.442% associated with CVD mortality. In the CKD population, CALLY values were negatively associated with all-cause and cardiovascular mortality, with BioAgeAccel significantly mediating this relationship.

## Introduction

Chronic Kidney Disease (CKD) represents a major global health challenge and is recognized as one of the leading causes of premature mortality worldwide [1]. This condition is marked by a progressive decline in renal function, frequently accompanied by systemic inflammation, malnutrition, and immune dysregulation, which contribute to numerous adverse health outcomes [2,3]. By combining markers of inflammation (C-reactive protein), nutritional status (albumin), and immune function (lymphocyte counts), the CALLY index offers a composite measure that may be invaluable in clinical assessments [4]. Initially, the CALLY index gained traction in oncology, demonstrating superior prognostic value compared to traditional biomarkers such as the neutrophil-to-lymphocyte ratio (NLR) and platelet-to-lymphocyte ratio (PLR) in cancer patients [5]. Recent studies have found that the CALLY index is negatively correlated with the prevalence of metabolic syndrome (MetS) and cardiorenal syndrome [6,7]. Previous studies using the NHANES database have confirmed the strong association of CALLY with mortality risk in the elderly population and in cancer populations. However, it has not been validated in the CKD population, and the overlapping mechanisms of CKD, MetS, and CVD highlight a possible association between the CALLY index and all-cause and CVD mortality in patients with CKD.

Moreover, the recent recognition of biological aging, assessed through indices such as Biological age acceleration (BioAgeAccel), has significantly influenced health outcomes [8,9]. Accelerated biological aging is associated with chronic inflammation, oxidative stress, and metabolic dysregulation [10–14], central to CKD progression. Recent studies highlighting the associations between biological aging and adverse health outcomes, including increased cardiovascular events and all-cause mortality [8,15,16], emphasize the necessity of investigating the mediating role of biological aging in the relationship between the CALLY index and mortality in CKD patients.

This study utilizes data from the National Health and Nutrition Examination Survey (NHANES) to investigate the association between the CALLY index and all-cause and cardiovascular disease mortality in patients with CKD and examine whether biological aging mediates these associations.

## Methods

### Data and study participants

**Figure 1.**
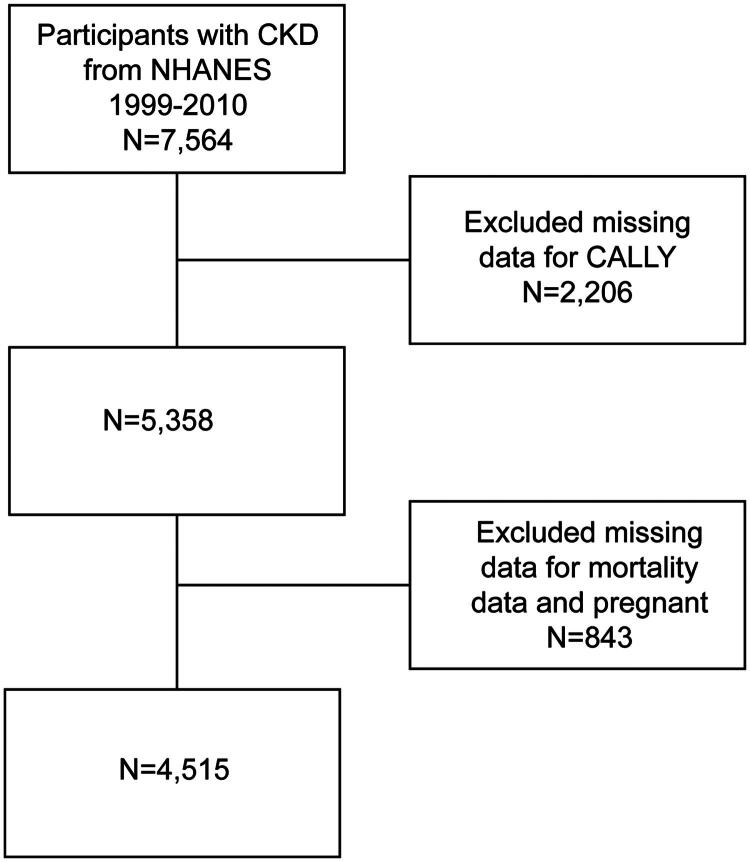
Flowchart of study participants. CALLY, C-reactive protein-albumin-lymphocyte; NHANES, National Health and Nutritional Examination Surveys; CKD, chronic kidney disease.

The National Health and Nutrition Examination Survey (NHANES) is a nationally representative study that collects health and nutrition data on the U.S. household population. Data were collected through a multistage probability sampling design, including structured household interviews, physical examinations at ambulatory centers, and laboratory tests. The NHANES protocol was approved by the Institutional Review Board (IRB) of the National Center for Health Statistics (NCHS), and all participants provided written informed consent. A total of 7,564 individuals with CKD were identified from the NHANES 1999–2010 dataset. We excluded 2,206 individuals due to missing albumin, lymphocyte, or C-reactive protein values required for the CALLY formula. Additionally, 802 individuals were excluded due to missing survival status. Finally, to ensure result reliability, 41 pregnant women were excluded. In total, 4,515 participants were included in the final analysis. The data screening process is illustrated in Figure 1. For the mediator BioAgeAccel, 175 participants had missing values. These individuals were excluded only from the mediation analysis, as their data were otherwise complete for the main analyses. This approach ensured that exclusion due to mediator missingness did not introduce unnecessary selection bias into the primary analyses. Missing values for other covariates were imputed using the random forest method. All data used in this study are publicly available at: https://www.cdc.gov/nchs/nhanes/?CDC_AAref_Val=https://www.cdc.gov/nchs/nhanes/index.htm.

### Definition of CKD

CKD was defined by the diagnostic criteria of the Kidney Disease: Improving Global Outcomes (KDIGO) guidelines. The estimated glomerular filtration rate (eGFR) was calculated using the updated Chronic Kidney Disease Epidemiology Collaboration (CKD-EPI) equation, incorporating serum creatinine, age, sex, and race. The albumin-to-creatinine ratio (ACR), expressed in mg/g, was obtained from spot morning urine samples and used to indicate microalbuminuria. Participants were considered to have CKD if they met either eGFR < 60 mL/min/1.73 m^2^ or ACR > 30 mg/g [17].

### Definition of the C-reactive protein-albumin-lymphocyte index

Blood count analysis, including neutrophils, platelets, monocytes, and lymphocytes, was performed using a Kurt HMX Hematology Analyzer, with results reported in standardized units (×10³ cells/μl). This study focuses on the CALLY Index, a novel metric derived from albumin levels, lymphocyte counts, and C-reactive protein (CRP) values. The CALLY Index is calculated as [(albumin, g/L) × (lymphocyte count, 10³ cells/μl)]/(CRP, mg/dL) and offers a comprehensive assessment of inflammatory, nutritional, and immune status.

### Outcome ascertainment

All-cause mortality was determined using National Death Index (NDI) records linked to the NHANES dataset, with follow-up through December 31, 2019. Cause-specific mortality was classified based on ICD-10 codes. Cardiovascular disease (CVD)-related deaths were defined by ICD-10 codes I00-I09, I11, I13, and I20-I51.The dataset is available at:https://ftp.cdc.gov/pub/Health_Statistics/NCHS/datalinkage/linked_mortality/.

### Measurement of BioAgeAccel

Biological age, as proposed by Klemera and Doubal [18], is derived from eight biomarkers: Ln-C reactive protein (CRP), serum creatinine, glycosylated hemoglobin, serum albumin, total cholesterol, blood urea nitrogen, alkaline phosphatase, and systolic blood pressure. The formula is given in Supplementary documents. BioAgeAccel is the value obtained by subtracting the actual age from the biological age.

### Assessment of covariates

Covariates included age, sex, race, education, marital status, poverty-to-income ratio (PIR), body mass index (BMI), smoking status, alcohol consumption, moderate activity, vigorous activity, diabetes, hypertension, hyperlipidemia, cardiovascular disease (CVD), estimated glomerular filtration rate (eGFR), alanine aminotransferase (ALT), aspartate aminotransferase (AST) and uric acid. Physical activity was classified as vigorous or moderate (yes/no). Participants who had smoked at least 100 cigarettes in their lifetime and were still smoking at the time of the survey were classified as current smokers. Ex-smokers were those who had smoked at least 100 cigarettes in their lifetime but had quit before the study. Individuals who had smoked fewer than 100 cigarettes in their lifetime were classified as nonsmokers. Nondrinkers were participants who had never consumed at least 12 alcoholic beverages in their lifetime or any single year. Participants who had consumed at least 12 alcoholic drinks in their lifetime or any single year but had abstained in the past 12 months were classified as ever drinkers. Current drinkers were those who had consumed at least 12 alcoholic beverages in their lifetime or any single year and had consumed alcohol in the past 12 months. Diabetes was defined as physician-diagnosed diabetes, a fasting blood glucose level ≥7.0 mmol/L, glycosylated hemoglobin ≥6.5%, a 75 g oral glucose tolerance test result ≥200 mg/dL, or glucose-lowering medication or insulin. Hypertension was defined as a history of hypertension, use of antihypertensive medication, or systolic blood pressure ≥130 mm Hg or diastolic blood pressure ≥80 mm Hg. Hyperlipidemia was defined as triglycerides ≥150 mg/dL, total cholesterol ≥200 mg/dL, low - density lipoprotein cholesterol (LDL-C) ≥ 130 mg/dL, high-density lipoprotein cholesterol (HDL-C) ≤ 40 mg/dL in men or ≤50 mg/dL in women, or use of lipid-lowering therapy. CVD was defined as a history of congestive heart failure, coronary artery disease, angina, stroke, or myocardial infarction.

### Statistical analysis

Missing covariate data were handled using a random‑forest–based imputation procedure (missForest), which accommodates mixed variable types and nonlinear relations [19]. The proportion of missingness for covariates was low (uric acid 0.02%, AST 0.42%, ALT 0.42%, BMI 2.79%, CVD 3.61%, smoking 3.70%, and PIR 8.82%); accordingly, single imputation with missForest was used for the main analyses [20,21]. This choice is supported by prior work showing strong performance of random‑forest imputation relative to common alternatives in biomedical datasets [22]. uACR was not imputed in the primary models because it is part of the KDIGO definition of CKD, and imputing it could lead to misclassification of disease status. Therefore, uACR was used only in sensitivity analyses among participants with available measurements. This strategy is consistent with current practice and acknowledges the risk of bias when imputing disease-defining variables [23,24]. Baseline characteristics were summarized as mean ± standard deviation (SD) for normally distributed continuous variables, median with interquartile range (IQR) for skewed continuous variables, and proportions for categorical variables. Between-group comparisons were conducted across tertiles of Ln-CALLY, using one-way analysis of variance (ANOVA) for normally distributed continuous variables, the Kruskal–Wallis test for skewed continuous variables, and the Chi-square test for categorical variables. Because the CALLY index shows marked right-skewness, especially driven by the CRP component, it was transformed using the natural logarithm (Ln-CALLY) prior to all regression analyses. This transformation reduces influence of extreme CRP values, stabilizes variance, and improves model fit and plausibility of log-linear assumptions in Cox regression, consistent with methods employed in prior studies using CALLY and similar composite biomarker indices [25–27]. Multivariate Cox regression assessed the association between CALLY and all-cause and CVD mortality. Three models were constructed: Model 1 (unadjusted); Model 2, adjusted for age, sex, and race; and Model 3, further adjusted for education, marital status, PIR, BMI, smoking status, alcohol consumption, moderate activity, vigorous activity, diabetes, hypertension, hyperlipidemia, CVD, eGFR, ALT, AST and uric acid. Subgroup analyses assessed the association between CALLY and all-cause mortality across different subgroups. We performed a Kaplan-Meier survival analysis to map the association of different Ln-CALLY groups with all-cause mortality and CVD mortality. Smoothed curve fitting and threshold effect analyses were performed to explore the nonlinear relationship between CALLY and all-cause or CVD mortality. Mediation analyses were conducted to examine the potential role of BioAgeAccel in mediating the association between Ln-CALLY and all-cause or CVD mortality, adjusting for covariates in Model 3, as per VanderWeele’s framework [28]. Previous studies have suggested that the atherogenic index of plasma (AIP) and the systemic immune-inflammation index (SII) are potential predictors of mortality in patients with CKD [29,30]. To further evaluate the predictive utility of CALLY, we compared its discrimination ability with AIP and SII for all-cause and cardiovascular mortality. Time-dependent ROC curves were constructed at 5-, 10-, and 15-year follow-up using the R package timeROC. The area under the curve (AUC) with 95% confidence intervals was calculated to quantify predictive performance. For the primary analyses, missing covariates were imputed using a random forest approach. Considering that uACR is a key variable in the definition of CKD and thus not suitable for imputation, we included it only in sensitivity analyses. Specifically, we repeated the Cox regression models with additional adjustment for uACR to assess the robustness of the findings. To reduce the potential influence of reverse causation, we excluded participants who died within the first two years of follow-up and reassessed associations using Cox models. In addition, to minimize the potential influence of extreme values, we conducted a sensitivity analysis by excluding participants in the lowest and highest 1% of the CALLY distribution. All statistical analyses were performed using R version 4.2.0 (The R Foundation for Statistical Computing, Vienna, Austria; http://www.R-project.org), and a two-tailed P value < 0.05 was considered statistically significant.

### Language editing

The manuscript was edited for readability, style, and grammatical accuracy using Grammarly, an artificial intelligence–based tool for non-generative copyediting.

## Results

### General characteristics of study participants

Table 1 presents the baseline characteristics of participants stratified by Ln-CALLY tertiles. No significant differences were found among the three groups in sex, marital status, and albuminuria (*p* > 0.05). However, significant differences were observed in BMI, diabetes mellitus, hypertension, race, smoking, and alcohol consumption (*p* < 0.05). The lower CALLY group had a higher proportion of individuals with eGFR < 60 mL/min/1.73 m^2^, BMI ≥ 30, and a greater prevalence of diabetes, hypertension, and CVD.

**Table 1.** Baseline characteristics of the study population.

	Tertile of Ln-CALLY			
Variables	T1	T2	T3	P-value
N	1505	1504	1506	
Age, years	64.642 ± 16.496	65.340 ± 16.944	60.075 ± 21.236	<0.001
BMI, kg/m²	31.639 ± 7.933	29.669 ± 6.209	26.527 ± 5.546	<0.001
PIR	2.194 ± 1.404	2.279 ± 1.411	2.411 ± 1.488	<0.001
Age, n (%)				<0.001
<60	417 (29.35%)	396 (27.87%)	542 (38.09%)	
> =60	1004 (70.65%)	1025(72.13%)	881 (61.91%)	
Sex, n (%)				0.213
Male	690 (45.847%)	702 (46.676%)	737 (48.938%)	
Female	815 (54.153%)	802 (53.324%)	769 (51.062%)	
Race, n (%)				0.002
Mexican American	252 (17.73%)	270 (19.00%)	240 (16.87%)	
Other Hispanic	77 (5.42%)	88 (6.19%)	93 (6.54%)	
Non-Hispanic White	748 (52.64%)	745 (52.43%)	760 (53.41%)	
Non-Hispanic Black	311 (21.89%)	269 (18.93%)	257 (18.06%)	
Other Race	33 (2.32%)	49 (3.45%)	73 (5.13%)	
Education, n (%)				<0.001
Under high school	604 (40.133%)	575 (38.231%)	489 (32.470%)	
Hight school or equivalent	334 (22.193%)	364 (24.202%)	353 (23.440%)	
College graduate or above	567 (37.674%)	565 (37.566%)	664 (44.090%)	
Marital Status, n (%)				0.813
Married or living with partner	813 (54.020%)	829 (55.120%)	827 (54.914%)	
Living alone	692 (45.980%)	675 (44.880%)	679 (45.086%)	
PIR, n (%)				<0.001
<1.3	511 (33.953%)	443 (29.455%)	424 (28.154%)	
> =1.3, <3.5	688 (45.714%)	749 (49.801%)	702 (46.614%)	
> =3.5	306 (20.332%)	312 (20.745%)	380 (25.232%)	
BMI, n (%)				<0.001
<25	272 (18.073%)	335 (22.274%)	644 (42.762%)	
> =25, <30	420 (27.907%)	512 (34.043%)	527 (34.993%)	
> =30	813 (54.020%)	657 (43.684%)	335 (22.244%)	
Smoke, n (%)				<0.001
Current smokers	281 (18.671%)	236 (15.691%)	231 (15.339%)	
Nonsmokers	665 (44.186%)	755 (50.199%)	842 (55.910%)	
Former smokers	559 (37.143%)	513 (34.109%)	433 (28.752%)	
Drink, n (%)				<0.001
Current drinkers	788 (52.359%)	794 (52.793%)	921 (61.155%)	
Nondrinkers	253 (16.811%)	249 (16.556%)	239 (15.870%)	
Former drinkers	464 (30.831%)	461 (30.652%)	346 (22.975%)	
Moderate activity, n (%)				<0.001
No	1035 (68.771%)	982 (65.293%)	906 (60.159%)	
Yes	470 (31.229%)	522 (34.707%)	600 (39.841%)	
Vigorous activity, n (%)				<0.001
No	1324 (87.973%)	1267 (84.242%)	1149 (76.295%)	
Yes	181 (12.027%)	237 (15.758%)	357 (23.705%)	
Diabetes, n (%)				<0.001
No	879 (58.405%)	962 (63.963%)	1070 (71.049%)	
Yes	626 (41.595%)	542 (36.037%)	436 (28.951%)	
Hypertension, n (%)				<0.001
No	270 (17.940%)	292 (19.415%)	474 (31.474%)	
Yes	1235 (82.060%)	1212 (80.585%)	1032 (68.526%)	
Hyperlipidemia, n (%)				<0.001
No	494 (32.824%)	490 (32.580%)	680 (45.153%)	
Yes	1011 (67.176%)	1014 (67.420%)	826 (54.847%)	
CVD, n (%)				<0.001
No	1020 (67.774%)	1108 (73.670%)	1166 (77.424%)	
Yes	485 (32.226%)	396 (26.330%)	340 (22.576%)	
UA, mg/dL	6.302 ± 1.813	6.046 ± 1.579	5.659 ± 1.580	<0.001
Creatinine, mg/dL	1.240 ± 1.065	1.164 ± 0.799	1.101 ± 0.796	<0.001
ALT, U/L	24.142 ± 29.159	24.645 ± 17.681	23.088 ± 16.534	0.137
AST, U/L	26.331 ± 19.094	26.452 ± 12.991	26.362 ± 15.989	0.978
eGFR, (ml/min/1.73m²)	69.874 ± 31.228	69.951 ± 28.376	76.655 ± 31.668	<0.001
eGFR, n (%)				0.003
≥60	746 (49.568%)	761 (50.598%)	833 (55.312%)	
<60	759 (50.432%)	743 (49.402%)	673 (44.688%)	
uACR, mg/g	268.742 ± 973.760	227.815 ± 814.154	182.187 ± 808.233	0.026
Albuminuria, n (%)				0.140
No	415 (28.700%)	470 (31.886%)	465 (31.292%)	
Yes	1031 (71.300%)	1004 (68.114%)	1021 (68.708%)	
BioAgeAccel	6.881 ± 20.126	−0.149 ± 19.349	−6.101 ± 18.471	<0.001
CALLY	81.250 (45.255–117.647)	290.593 (221.740–377.357)	1050.000 (711.111–1771.339)	<0.001
Follow-up time, months	111.805 ± 62.959	123.094 ± 57.515	130.144 ± 55.663	<0.001
All-cause mortality, n (%)				<0.001
No	623 (41.395%)	727 (48.338%)	901 (59.827%)	
Yes	882 (58.605%)	777 (51.662%)	605 (40.173%)	
CVD mortality, n (%)				<0.001
No	1233 (81.927%)	1264 (84.043%)	1318 (87.517%)	
Yes	272 (18.073%)	240 (15.957%)	188 (12.483%)	

Baseline characteristics of NHANES participants, 1999–2010. Categorized according to CALLY tertiles. The baseline characteristics table presented continuous variables as means with standard deviation (SD) or median with interquartile range (IQR), while categorical variables were reported as proportions. Group comparisons were performed using one-way analysis of variance (ANOVA) for normally distributed continuous variables and the Kruskal–Wallis test for skewed continuous variables. The Chi-square test was applied to compare categorical variables.

CALLY, C-reactive protein-albumin-lymphocyte; BMI, body mass index; CKD, chronic kidney disease; BMI, body mass index; PIR, poverty income ratio; CVD, cardiovascular disease; eGFR, estimated glomerular filtration rate; uACR, urine albumin - creatinine ratio; ALT, alanine aminotransferase; AST, aspartate aminotransferase; BioAgeAccel, Biological Age Acceleration.

### Association between CALLY and mortality

As shown in Table 2, higher Ln-CALLY was significantly associated with a reduced risk of all-cause mortality in Model 1 (HR = 0.818, 95% CI: 0.793–0.844). The association remained consistent after multivariable adjustment in Model 2 (HR = 0.827, 95% CI: 0.801–0.855) and Model 3 (HR = 0.845, 95% CI: 0.817–0.874). Similarly, for cardiovascular mortality, a significant inverse association was observed in Model 1 (HR = 0.825, 95% CI: 0.780–0.872), Model 2 (HR = 0.839, 95% CI: 0.790–0.890), and Model 3 (HR = 0.860, 95% CI: 0.809–0.915). When participants were stratified into tertiles of Ln-CALLY, those in the third tertile had a 37.4% lower risk of all-cause mortality and a 34.0% lower risk of cardiovascular mortality compared with the first tertile. A significant linear trend was consistently observed across tertiles (P for trend < 0.001).

**Table 2.** Association of Ln-CALLY with mortality in the CKD population.

	Model 1	Model 2	Model 3
	HR 95% CI	HR 95% CI	HR 95% CI
**All-cause mortality**	0.818 (0.793, 0.844)	0.827 (0.801, 0.855)	0.845 (0.817, 0.874)
Ln-CALLY			
T1	Ref	Ref	Ref
T2	0.795 (0.722, 0.875)	0.713 (0.647, 0.785)	0.756 (0.685, 0.834)
T3	0.581 (0.523, 0.644)	0.592 (0.533, 0.657)	0.626 (0.562, 0.698)
*P* for trend	<0.001	<0.001	<0.001
			
**Cardiovascular mortality**	0.825 (0.780, 0.872)	0.839 (0.790, 0.890)	0.860 (0.809, 0.915)
Ln-CALLY			
T1	Ref	Ref	Ref
T2	0.800 (0.672, 0.951)	0.724 (0.608, 0.862)	0.785 (0.658, 0.937)
T3	0.588 (0.488, 0.708)	0.606 (0.503, 0.731)	0.660 (0.542, 0.803)
*P* for trend	<0.001	<0.001	<0.001

HR: hazard ratio.

95% CI: 95% confidence interval.

Model 1: no covariates were adjusted.

Model 2: Adjusted for age, sex, and race.

Model 3: Adjusted for age, sex, race, education, marital status, PIR, body mass index, smoking, drinking, moderate activity, vigorous activity, diabetes, hypertension, hyperlipidemia, cardiovascular disease, eGFR, ALT, AST and uric acid.

**Figure 2.**
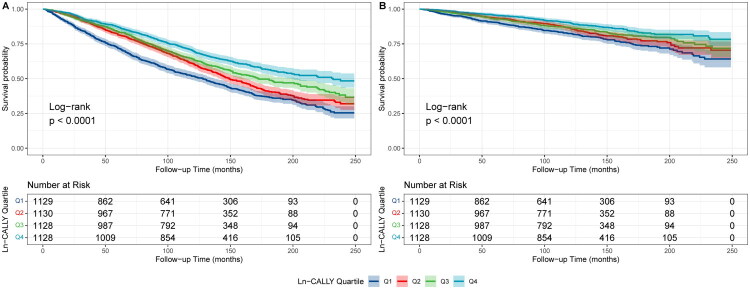
Kaplan–Meier survival curves for the effect of CALLY on long-term all-cause mortality (A) and cardiovascular disease mortality (B) in a CKD population.

The Kaplan-Meier curve (Figure 2) supports these findings. The curve illustrates that CKD patients with lower Ln-CALLY levels had a greater decline in survival over follow-up compared to those with higher Ln-CALLY levels.

**Figure 3.**
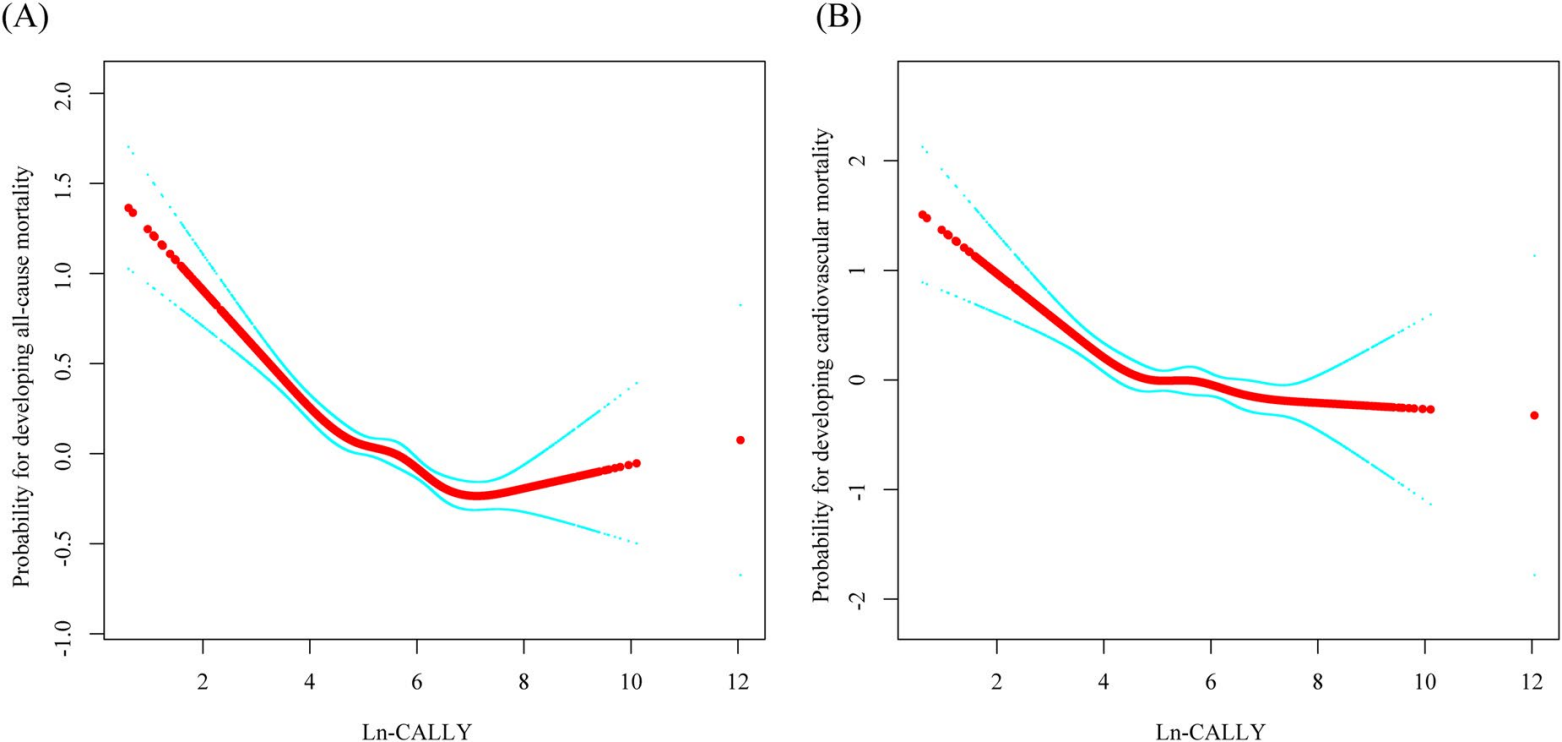
Nonlinear associations of ln-CALLY with all-cause (A) and cardiovascular (B) mortality in CKD.

As shown in Table 3 and Figure 3, nonlinear associations were observed between Ln-CALLY and both all-cause and cardiovascular mortality among individuals with CKD. Using a standard linear model, higher Ln-CALLY was inversely associated with all-cause mortality (HR = 0.845, 95% CI: 0.817–0.874, *p* < 0.001) and cardiovascular mortality (HR = 0.860, 95% CI: 0.809–0.915, *p* < 0.001). A two-piecewise linear regression model further identified an inflection point at Ln-CALLY = 4.96 (CALLY = 142.59). Below this threshold, Ln-CALLY was strongly associated with decreased risk of all-cause mortality (HR = 0.750, 95% CI: 0.698–0.806, *p* < 0.001) and cardiovascular mortality (HR = 0.742, 95% CI: 0.650–0.848, *p* < 0.001). In contrast, above the threshold, the associations were weaker for all-cause mortality (HR = 0.912, 95% CI: 0.864–0.962, *p* < 0.001) and became non-significant for cardiovascular mortality (HR = 0.942, 95% CI: 0.856–1.036, *p* = 0.219). Log-likelihood ratio tests supported the nonlinear relationship for both outcomes (*p* < 0.001 for all-cause mortality; *p* = 0.019 for cardiovascular mortality).

**Table 3.** Threshold effect analysis of CALLY with mortality.

	Adjusted HR (95% CI), P-Value
**CALLY vs All-cause mortality**	
Fitting by the standard linear model	0.845 (0.817, 0.874) <0.001
Fitting by the two-piecewise linear model	
Ln-CALLY	
Inflection point	4.96
Ln-CALLY < 4.96	0.750 (0.698, 0.806) <0.001
Ln-CALLY > 4.96	0.912 (0.864, 0.962) <0.001
Log likelihood ratio	<0.001
**CALLY vs CVD mortality**	0.860 (0.809, 0.915) <0.001
Fitting by the standard linear model	
Fitting by the two-piecewise linear model	
Ln-CALLY	
Inflection point	4.96
Ln-CALLY < 4.96	0.742 (0.650, 0.848) <0.001
Ln-CALLY > 4.96	0.942 (0.856, 1.036) 0.219
Log likelihood ratio	0.019

HR: hazard ratio.

95% CI: 95% confidence interval.

Adjusted for age, sex, race, education, marital status, PIR, body mass index, smoking, drinking, moderate activity, vigorous activity, diabetes, hypertension, hyperlipidemia, cardiovascular disease, eGFR, ALT, AST and uric acid.

Adjusted for age, sex, race, education, marital status, PIR, body mass index, smoking, drinking, moderate activity, vigorous activity, diabetes, hypertension, hyperlipidemia, cardiovascular disease, eGFR, ALT, AST and uric acid.

### Subgroup analysis

To evaluate the robustness of the findings, subgroup analyses were conducted across multiple strata. As shown in Table 4, the inverse associations of Ln-CALLY with all-cause and cardiovascular mortality were generally consistent across sex, marital status, PIR, BMI, smoking, drinking, diabetes, hypertension, hyperlipidemia, CVD, physical activity, and eGFR. Although not all subgroup estimates reached statistical significance, no significant interactions were detected (P for interaction > 0.05).

**Table 4.** Subgroup analysis of the relationship between Ln-CALLY and all-cause and cardiovascular mortality.

Character	All-cause mortality			Cardiovascular mortality		
	HR (95% CI)	*P*-value	P for interaction	HR (95% CI)	*P*-value	P for interaction
Sex			0.103			0.237
Male	0.823 (0.786, 0.862)	<0.001		0.832 (0.766, 0.904)	<0.001	
Female	0.869 (0.828, 0.913)	<0.001		0.894 (0.818, 0.978)	0.015	
Education			0.653			0.129
Under high school	0.857 (0.814, 0.903)	<0.001		0.907 (0.825, 0.997)	0.042	
Hight school or equivalent	0.850 (0.792, 0.912)	<0.001		0.891 (0.785, 1.013)	0.077	
College graduate or above	0.828 (0.784, 0.875)	<0.001		0.794 (0.719, 0.877)	<0.001	
Marital Status,			0.165			0.274
Married or living with partner	0.814 (0.777, 0.854)	<0.001		0.832 (0.764, 0.906)	<0.001	
Living alone	0.876 (0.835, 0.918)	<0.001		0.890 (0.816, 0.970)	0.008	
PIR			0.967			0.158
<1.3	0.850 (0.801, 0.902)	<0.001		0.912 (0.818, 1.017)	0.097	
> =1.3, <3.5	0.843 (0.804, 0.885)	<0.001		0.870 (0.798, 0.949)	0.002	
> =3.5	0.840 (0.780, 0.905)	<0.001		0.772 (0.676, 0.881)	0.000	
BMI			0.156			0.274
<25	0.874 (0.827, 0.923)	<0.001		0.760 (0.696, 0.830)	<0.001	
> =25, <30	0.849 (0.801, 0.900)	<0.001		0.814 (0.737, 0.899)	<0.001	
> =30	0.805 (0.756, 0.858)	<0.001		0.851 (0.763, 0.949)	0.004	
Smoke			0.118			0.293
Current smokers	0.790 (0.723, 0.864)	<0.001		0.806 (0.678, 0.958)	0.015	
Nonsmokers	0.832 (0.790, 0.877)	<0.001		0.831 (0.759, 0.910)	<0.001	
Former smokers	0.874 (0.831, 0.918)	<0.001		0.908 (0.828, 0.996)	0.040	
Drink			0.251			0.242
Current drinkers	0.821 (0.782, 0.863)	<0.001		0.787 (0.719, 0.862)	<0.001	
Nondrinkers	0.847 (0.784, 0.915)	<0.001		0.936 (0.819, 1.070)	0.332	
Former drinkers	0.874 (0.827, 0.924)	<0.001		0.915 (0.827, 1.013)	0.087	
Moderate activity			0.073			0.192
No	0.861 (0.828, 0.897)	<0.001		0.895 (0.833, 0.962)	<0.001	
Yes	0.806 (0.759, 0.857)	<0.001		0.774 (0.691, 0.867)	<0.001	
Vigorous activity			0.887			0.785
No	0.844 (0.815, 0.874)	<0.001		0.858 (0.805, 0.915)	<0.001	
Yes	0.852 (0.756, 0.960)	0.008		0.887 (0.707, 1.112)	0.297	
Diabetes			0.859			0.479
No	0.843 (0.806, 0.881)	<0.001		0.845 (0.781, 0.914)	<0.001	
Yes	0.848 (0.805, 0.892)	<0.001		0.883 (0.803, 0.971)	0.011	
Hypertension, n(%)			0.155			0.325
No	0.799 (0.736, 0.869)	<0.001		0.804 (0.695, 0.931)	0.004	
Yes	0.854 (0.823, 0.885)	<0.001		0.872 (0.815, 0.933)	<0.001	
Hyperlipidemia			0.853			0.381
No	0.842 (0.800, 0.886)	<0.001		0.835 (0.762, 0.914)	<0.001	
Yes	0.847 (0.810, 0.885)	<0.001		0.881 (0.812, 0.955)	0.002	
CVD			0.501			0.059
No	0.874 (0.837, 0.912)	<0.001		0.907 (0.835, 0.986)	0.022	
Yes	0.803 (0.762, 0.846)	<0.001		0.809 (0.740, 0.884)	<0.001	
eGFR			0.068			0.649
≥60	0.879 (0.832, 0.929)	<0.001		0.939 (0.848, 1.039)	0.222	
<60	0.825 (0.791, 0.860)	<0.001		0.821 (0.760, 0.886)	<0.001	

### The mediating role of BioAgeAccel

As shown in Supplementary Table 1, Ln-CALLY was inversely associated with BioAgeAccel across all three models. In the fully adjusted model (Model 3), the regression coefficient was β = −3.364 (95% CI: −3.750, −2.979, *p* < 0.001). Supplementary Table 2 further demonstrated that BioAgeAccel was positively asso­ciated with both all-cause mortality (HR = 1.010, 95% CI: 1.008–1.012, *p* < 0.001) and CVD mortality (HR = 1.014, 95% CI: 1.010–1.018, *p* < 0.001).

**Figure 4.**
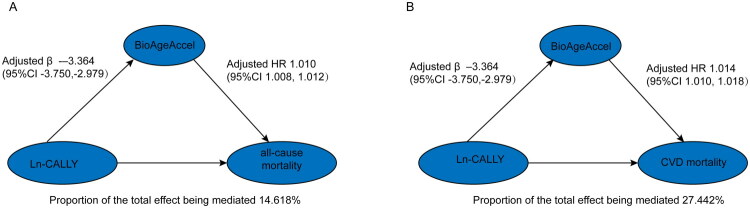
Mediation analysis of the association between Ln-CALLY and mortality through BioAgeAccel. (A) Mediation effect of BioAgeAccel on the association between Ln-CALLY and all-cause mortality. (B) Mediation effect of BioAgeAccel on the association between Ln-CALLY and cardiovascular mortality. All models were adjusted for age, sex, race, education, marital status, PIR, body mass index, smoking, drinking, moderate and vigorous physical activity, diabetes, hypertension, hyperlipidemia, cardiovascular disease, eGFR, ALT, AST, and uric acid.

Mediation analyses (Figure 4 and Table 5) revealed that BioAgeAccel partially mediated the associations of Ln-CALLY with mortality outcomes. Specifically, BioAgeAccel explained 14.618% (95% CI: 0.091–0.219, *p* < 0.001) of the association between Ln-CALLY and all-cause mortality, and 27.442% (95% CI: 0.148–0.505, *p* < 0.001) of the association with CVD mortality. Both the mediation and direct effects remained statistically significant after adjustment for potential confounders.

**Table 5.** The mediating role of BioAgeAccel in the association between CALLY and all-cause mortality and CVD mortality.

Mediation effect	Estimate	95% CI lower	95% CI upper	*P*-value
All-cause mortality				
Total effect	78.363	60.496	97.500	<0.001
Mediation effect	11.475	7.273	16.200	<0.001
Direct effect	66.888	49.196	85.890	<0.001
Proportion mediated	0.146	0.091	0.219	<0.001
CVD mortality				
Total effect	215.938	116.601	346.607	<0.001
Mediation effect	59.528	33.371	94.035	<0.001
Direct effect	156.410	61.099	270.105	0.002
Proportion mediated	0.274	0.148	0.505	<0.001

Adjusted for age, sex, race, education, marital status, PIR, body mass index, smoking, drinking, moderate activity, vigorous activity, diabetes, hypertension, hyperlipidemia, cardiovascular disease, eGFR, ALT, AST and uric acid.

### Predictive performance of CALLY compared with AIP and SII

**Figure 5.**
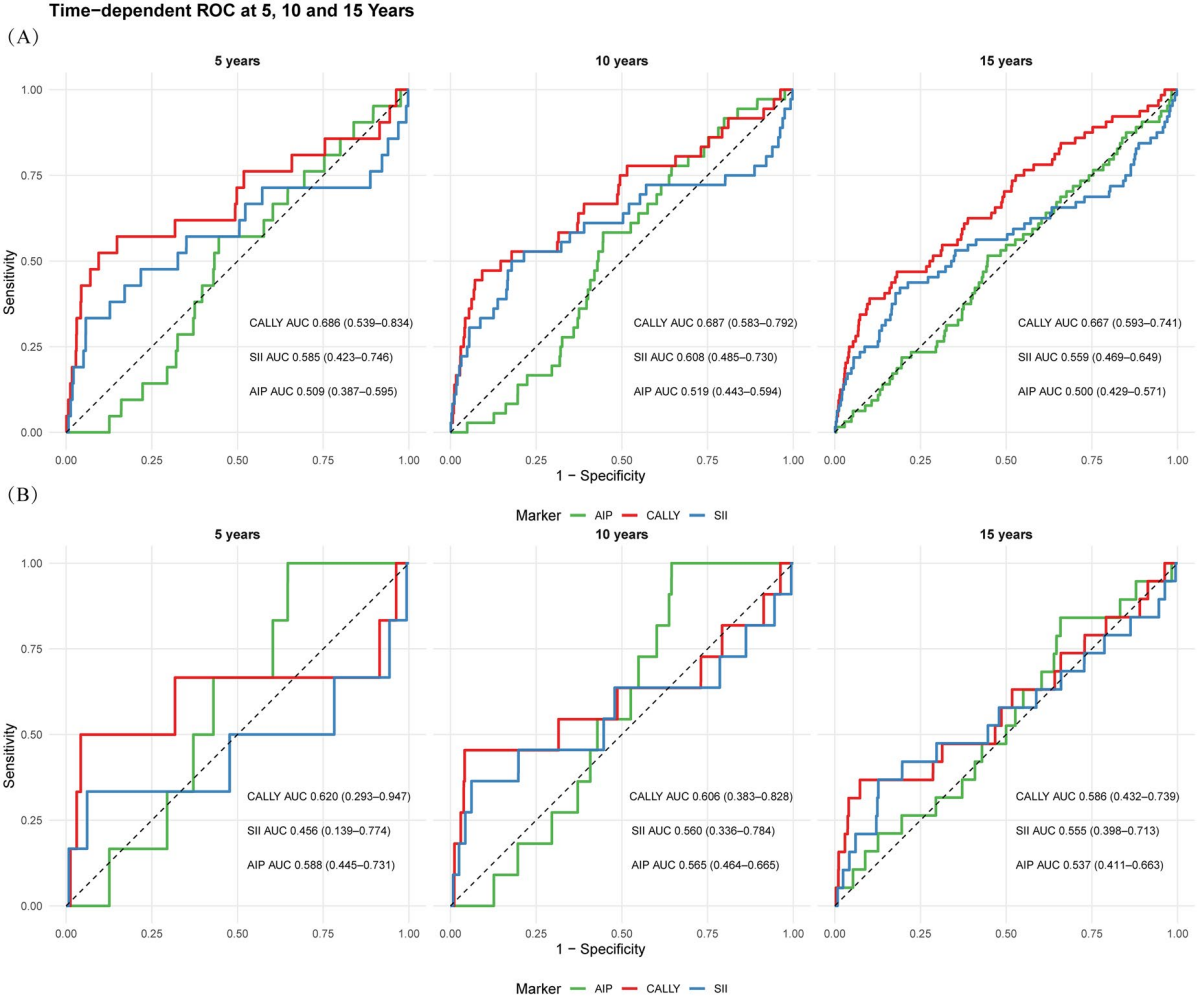
Time-dependent ROC analysis comparing the predictive performance of CALLY, AIP, and SII for mortality in patients with CKD. (A) Predictive ability for all-cause mortality at 5, 10, and 15 years. (B) Predictive ability for cardiovascular mortality at 5, 10, and 15 years. The area under the curve (AUC) with 95% confidence intervals is presented for each marker.

As shown in Figure 5, CALLY consistently demonstrated superior predictive performance for both all-cause and cardiovascular mortality in CKD compared with AIP and SII. For all-cause mortality (Figure 5(A)), the AUCs of CALLY were 0.686 (95% CI: 0.539–0.834), 0.687 (95% CI: 0.583–0.792), and 0.667 (95% CI: 0.593–0.741) at 5, 10, and 15 years, respectively, which were higher than those of AIP and SII. Similarly, for cardiovascular mortality (Figure 5(B)), CALLY achieved AUCs of 0.620 (95% CI: 0.293–0.947), 0.606 (95% CI: 0.383–0.828), and 0.568 (95% CI: 0.432–0.739) at 5, 10, and 15 years, again outperforming AIP and SII. These findings suggest that CALLY may provide better long-term prognostic value for mortality risk stratification in CKD patients.

### Sensitivity analysis

In the sensitivity analysis, after additional adjustment for the urine albumin-to-creatinine ratio (uACR) (Supplementary Table 3), the inverse association between log-transformed CALLY and all-cause and cardiovascular mortality remained essentially unchanged. Furthermore, after excluding participants who died within the first 2 years of follow-up, we conducted an additional Cox regression analysis (Supplementary Table 4). The inverse association remained consistent. In addition, when extreme values of CALLY were truncated in sensitivity analyses (Supplementary Table 5), the associations were materially unchanged, further supporting the robustness of our findings.

## Discussion

This study showed that a lower CALLY index was significantly linked to a higher risk of all-cause and cardiovascular disease (CVD) mortality in patients with chronic kidney disease (CKD). This association was particularly pronounced when Ln-CALLY was below 4.96 and remained consistent across subgroups, irrespective of eGFR levels. Furthermore, BioAgeAccel partially mediated this association, contributing 14.618% to the effect on all-cause mortality and 27.442% on CVD mortality.

Growing evidence supports the prognostic value of the CALLY index across diseases. It has been associated with outcomes in rectal, liver, esophageal, and lung cancers [5,31–34], and with a lower risk of severe coronary artery disease in patients with STEMI [35]. More recently, lower CALLY values predicted prolonged hospitalization in patients with heart failure [36]. Similarly, systemic inflammatory–nutritional indices, such as the SII, have been linked to the severity of stable coronary artery disease [37], underscoring the broader utility of such markers in cardiovascular risk assessment. Consistent with a cohort study in older U.S. adults [38], our findings extend these associations to CKD patients and reveal a nonlinear relationship between Ln-CALLY and mortality. We identified an inflection point at Ln-CALLY ≈ 4.96, corresponding to a raw CALLY value of approximately 142.6. Below this threshold, small decreases in Ln-CALLY are associated with disproportionately larger increases in mortality risk, consistent with models in which low inflammatory–nutritional–immune reserve leads to rapid decompensation. Above this point, risk increases more gradually, suggesting a plateau effect. While this threshold is data-driven and exploratory, it may reflect the level at which the combined effect of inflammation, malnutrition, and immune suppression begins to overcome host compensatory capacity. As with other well-known biomarkers (e.g. hs-CRP >3 mg/L; albuminuria ACR ≥30 mg/g), such cutoffs may assist in risk stratification [39–41]. Prospective validation in diverse CKD cohorts is needed to confirm whether Ln-CALLY = 4.96 defines a clinically actionable threshold.

Furthermore, our study further explored the mediating role of biological aging in this association. Biological age acceleration has been increasingly implicated as a downstream consequence of chronic inflammation and oxidative stress in CKD, and relates to both kidney function decline and adverse outcomes. Recent work indicates that epigenetic age acceleration both influences and reflects kidney function, capturing CKD-related ‘premature aging.’ Dialysis and transplantation studies further suggest altered trajectories of epigenetic aging in advanced disease [42,43]. Integrating BioAgeAccel thus provides a mechanistic bridge linking CALLY (inflammation–nutrition–immune status) to mortality risk. More broadly, validated epigenetic/phenotypic aging measures (e.g. DNAm PhenoAge, GrimAge) predict mortality across populations, supporting the plausibility that biology encoded in “aging clocks” intersects with CALLY-reflected pathways [44,45]. In CKD, chronic inflammation activates pro-inflammatory pathways, including nuclear factor-kappa B (NF-κB) and interleukin-6 (IL-6) signaling, driving oxidative stress and endothelial dysfunction [46,47]. These mechanisms hasten atherosclerosis and cardiovascular events, accounting for the strong link between low CALLY Index values and CVD mortality. Furthermore, malnutrition, indicated by low albumin levels, contributes to muscle wasting, impaired immune responses, and heightened infection susceptibility, elevating all-cause mortality risk [48]. Our mediation analyses indicate that BioAgeAccel partially mediates the relationship between the CALLY Index and mortality.

Elevated CRP is a significant marker for systemic inflammation and is linked to oxidative stress, which can damage various cellular components, including DNA, proteins, and lipids Fay [49,50]. This oxidative damage accelerates cellular senescence, characterized as an irreversible cell cycle arrest. It triggers the release of pro-inflammatory cytokines and matrix-degrading enzymes, collectively termed senescence-associated secretory phenotypes (SASPs) [51,52]. SASPs contribute to tissue dysfunction and organ damage, particularly within the cardiovascular system, thereby exacerbating atherosclerosis, vascular calcification, and endothelial dysfunction [53].

Low albumin levels reflect nutritional deficiencies associated with increased oxidative stress and compromised antioxidant defenses. Albumin is an important antioxidant in plasma, effectively scavenging reactive oxygen species (ROS) and protecting cells from oxidative damage [54]. In patients with chronic kidney disease, hypoalbuminemia can exacerbate oxidative stress, further accelerating cellular senescence and biological aging. This phenomenon is particularly prevalent in hemodialysis patients [55].

Moreover, chronic inflammation coupled with oxidative stress can impair lymphocyte proliferation and function, leading to immune senescence, a decline in immune system efficiency associated with aging [56]. This immune senescence results in an accumulation of dysfunctional T cells, significantly diminishing the body’s ability to respond effectively to infections and malignancies, consequently heightening the risk of all-cause mortality [25,57]. In this context, immune senescence perpetuates chronic inflammation through the persistent secretion of pro-inflammatory cytokines, creating a detrimental feedback loop that exacerbates biological aging [58–60].

Building on these findings, the CALLY index could serve as a simple and cost-effective marker for mortality risk stratification in CKD. Future prospective and interventional studies are needed to validate its thresholds and to test whether modifying inflammation, nutrition, or immune function can alter outcomes. This study has several notable strengths. Leveraging a large, nationally representative cohort from NHANES, we systematically examined the association of the CALLY index with all-cause and cardiovascular mortality in patients with CKD. To our knowledge, this is the first study to evaluate the mediating role of biological age acceleration in this context. The use of rigorous subgroup and sensitivity analyses further enhanced the robustness of the findings.

Time-dependent AUCs for CALLY ranged from 0.586 to 0.687. According to established guidelines, values between 0.60–0.75 represent possibly helpful discrimination [61]; thus most horizons achieved this level, while the 15-year CVD AUC (0.586) was borderline. Although modest, these results are clinically relevant given that CALLY was evaluated as a single biomarker. In practice, its predictive performance would likely improve when combined with traditional risk factors such as age, kidney function, and comorbidities within multivariable models. In line with the exploratory aim of this study, we focused on the independent prognostic value of CALLY, but future work should assess its incremental utility and clinical benefit in comprehensive CKD risk frameworks.

This study also has several limitations. First, the CALLY index and its components were measured only at baseline; thus, we could not account for longitudinal changes during follow-up. Biomarkers related to inflammation, nutrition, and immune function fluctuate over time, especially in CKD patients, and reliance on a single measurement may introduce exposure misclassification and regression-dilution bias, potentially attenuating the true associations observed. Future studies incorporating repeated measurements or time-updated modeling are needed to reflect temporal dynamics better. Second, 2,206 individuals were excluded due to missing CALLY components. Although other covariates were imputed using a random forest method, excluding these participants could have introduced selection bias if the missingness was not completely random. Statistical approaches such as inverse-probability weighting may help mitigate this limitation in future analyses. Third, residual confounding cannot be entirely ruled out despite adjustment for a wide range of confounders. Factors related to CKD progression and treatment, such as renin–angiotensin–aldosterone system inhibitors, dialysis initiation, or nutritional interventions, may influence both the CALLY index and mortality risk but were unavailable in NHANES. Moreover, reverse causality cannot be entirely excluded because the CALLY index and biomarkers were measured only once. Fourth, the study population was derived from NHANES, which is designed to represent non-institutionalized U.S. adults. As a result, the findings may not be generalizable to populations with different ethnic, socioeconomic, and healthcare backgrounds, particularly in Asia or low- and middle-income countries. Differences in demographic profiles, lifestyle, and clinical care practices may modify both the predictive value of the CALLY index and its interpretation. Notably, the mediating role of biological age acceleration in the association between CALLY and mortality was demonstrated only in a U.S. population. Whether this pathway is consistent in non-Western populations remains uncertain and requires external validation. Future longitudinal studies in diverse cohorts are warranted to confirm the observed associations’ robustness and clarify the role of biological aging across different settings. Finally, multiple subgroups and sensitivity analyses increase the potential risk of inflated type I error. Although our primary hypotheses were prespecified and the main findings were consistent across analyses, future research may consider statistical approaches such as false-discovery-rate control to minimize this concern further.

## Conclusions

In conclusion, this study demonstrated a strong association between the CALLY index and all-cause and CVD mortality among CKD patients in the U.S. A nonlinear relationship was observed, with particularly pronounced effects at Ln-CALLY values below 4.96.Additionally, biological age acceleration was identified as a significant partial mediator in this relationship.

### Clinical trial number

Not applicable.

## Supplementary Material

Supplementary Table 4.docx

Supplementary Table 3.docx

Supplementary documents.docx

Supplementary Table 5.docx

Supplementary Table 1.docx

Figure legends.docx

Supplementary Table 2.docx

## Data Availability

The data used in this study were obtained from the National Health and Nutrition Examination Survey (NHANES), a publicly available dataset maintained by the Centers for Disease Control and Prevention (CDC). The data can be accessed at: https://www.cdc.gov/nchs/nhanes/?CDC_AAref_Val=https://www.cdc.gov/nchs/nhanes/index.htm
