# Supplementary material for: Negative association of C-reactive protein-albumin-lymphocyte index (CALLY index) with all-cause and cardiovascular mortality in population with CKD: the mediating role of biological age acceleration
Source: Ren Fail. 2025 Nov 18;47(1):2586892. doi: 10.1080/0886022X.2025.2586892 (PMC12632228; doi:10.1080/0886022X.2025.2586892)
Supplement: Supplementary Table 4.docx [file IRNF_A_2586892_SM5514.docx]

**Supplementary Table 4.** Sensitivity analysis of the association between Ln-CALLY and mortality in the CKD population excluding deaths within two years.

|  | Model 1 | **Model** 2 | **Model** 3 |
| --- | --- | --- | --- |
|  | **HR** 95% CI | **HR** 95% CI | **HR** 95% CI |
| **All-cause mortality** | 0.846 (0.818, 0.875) | 0.857 (0.827, 0.889) | 0.873 (0.841, 0.906) |
| Ln-CALLY |  |  |  |
| T1 | Ref | Ref | Ref |
| T2 | 0.828 (0.746, 0.918) | 0.760 (0.685, 0.843) | 0.801 (0.720, 0.890) |
| T3 | 0.615 (0.550, 0.687) | 0.633 (0.566, 0.708) | 0.671 (0.597, 0.754) |
| *P* for trend | <0.001 | <0.001 | <0.001 |
|  |  |  |  |
| **Cardiovascular mortality** | 0.863 (0.812, 0.918) | 0.881 (0.825, 0.940) | 0.900 (0.840, 0.963) |
| Ln-CALLY |  |  |  |
| T1 | Ref | Ref | Ref |
| T2 | 0.839 (0.695, 1.012) | 0.775 (0.642, 0.936) | 0.834 (0.688, 1.010) |
| T3 | 0.615 (0.502, 0.752) | 0.638 (0.521, 0.782) | 0.689 (0.557, 0.853) |
| *P* for trend | <0.001 | <0.001 | <0.001 |

HR: hazard ratio

95% CI: 95% confidence interval

Model 1: no covariates were adjusted

Model 2: Adjusted for age, sex, and race

Model 3:Adjusted for age, sex, race, education, marital status, PIR, body mass index, smoking, drinking, moderate activity, vigorous activity, diabetes, hypertension, hyperlipidemia, cardiovascular disease, eGFR, ALT, AST and uric acid.
