# Supplementary material for: Negative association of C-reactive protein-albumin-lymphocyte index (CALLY index) with all-cause and cardiovascular mortality in population with CKD: the mediating role of biological age acceleration
Source: Ren Fail. 2025 Nov 18;47(1):2586892. doi: 10.1080/0886022X.2025.2586892 (PMC12632228; doi:10.1080/0886022X.2025.2586892)
Supplement: Supplementary Table 3.docx [file IRNF_A_2586892_SM5513.docx]

**Supplementary Table 3.** Sensitivity analysis of the association between Ln-CALLY and mortality in the CKD population after additional adjustment for uACR.

|  | Model 1 | **Model** 2 | **Model** 3 |
| --- | --- | --- | --- |
|  | **HR** 95% CI | **HR** 95% CI | **HR** 95% CI |
| **All-cause mortality** | 0.827 (0.801, 0.854) | 0.837 (0.809, 0.866) | 0.860 (0.830, 0.890) |
| Ln-CALLY |  |  |  |
| T1 | Ref | Ref | Ref |
| T2 | 0.816 (0.739, 0.901) | 0.734 (0.665, 0.811) | 0.790 (0.714, 0.873) |
| T3 | 0.595 (0.535, 0.661) | 0.607 (0.545, 0.675) | 0.659 (0.590, 0.737) |
| *P* for trend | <0.001 | <0.001 | <0.001 |
|  |  |  |  |
| **Cardiovascular mortality** | 0.832 (0.786, 0.881) | 0.847 (0.797, 0.900) | 0.875 (0.821, 0.932) |
| Ln-CALLY |  |  |  |
| T1 | Ref | Ref | Ref |
| T2 | 0.821 (0.688, 0.980) | 0.746 (0.625, 0.891) | 0.823 (0.687, 0.987) |
| T3 | 0.601 (0.497, 0.726) | 0.620 (0.512, 0.750) | 0.696 (0.569, 0.851) |
| *P* for trend | <0.001 | <0.001 | <0.001 |

Model 1: no covariates were adjusted

Model 2: Adjusted for age, sex, and race

Model 3:Adjusted for age, sex, race, education, marital status, PIR, body mass index, smoking, drinking, moderate activity, vigorous activity, diabetes, hypertension, hyperlipidemia, cardiovascular disease, eGFR, ALT, AST and uric acid.

HR: hazard ratio; 95% CI: 95% confidence interval; ref, reference.
