# Supplementary material for: Negative association of C-reactive protein-albumin-lymphocyte index (CALLY index) with all-cause and cardiovascular mortality in population with CKD: the mediating role of biological age acceleration
Source: Ren Fail. 2025 Nov 18;47(1):2586892. doi: 10.1080/0886022X.2025.2586892 (PMC12632228; doi:10.1080/0886022X.2025.2586892)
Supplement: Supplementary documents.docx [file IRNF_A_2586892_SM5512.docx]

Supplementary information: Calculation of biological age


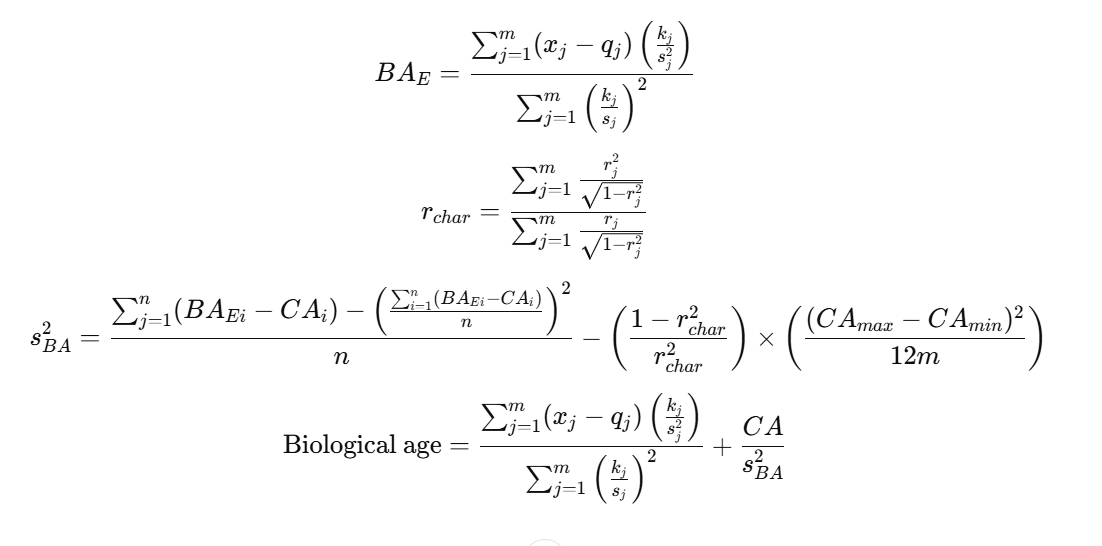


j and i denote the number of biomarkers and samples, respectively. The values k, q and s are the regression slope, intercept and root mean square error of the biomarker regressed on actual age, respectively. The value r2 indicates the variance explained by the regression of the biomarker on actual age.
