# Supplementary material for: Negative association of C-reactive protein-albumin-lymphocyte index (CALLY index) with all-cause and cardiovascular mortality in population with CKD: the mediating role of biological age acceleration
Source: Ren Fail. 2025 Nov 18;47(1):2586892. doi: 10.1080/0886022X.2025.2586892 (PMC12632228; doi:10.1080/0886022X.2025.2586892)
Supplement: Supplementary Table 5.docx [file IRNF_A_2586892_SM5511.docx]

**Supplementary Table 5.** Sensitivity analysis of the association between Ln-CALLY and mortality in the CKD population after excluding the top and bottom 1 % of CALLY values.

|  | Model 1 | **Model** 2 | **Model** 3 |
| --- | --- | --- | --- |
|  | **HR** 95% CI | **HR** 95% CI | **HR** 95% CI |
| **All-cause mortality** | 0.826 (0.798, 0.854) | 0.829 (0.800, 0.859) | 0.847 (0.816, 0.879) |
| Ln-CALLY |  |  |  |
| T1 | Ref | Ref | Ref |
| T2 | 0.804 (0.729, 0.886) | 0.726 (0.658, 0.801) | 0.774 (0.701, 0.855) |
| T3 | 0.605 (0.546, 0.672) | 0.604 (0.544, 0.671) | 0.644 (0.577, 0.719) |
| *P* for trend | <0.001 | <0.001 | <0.001 |
|  |  |  |  |
| **Cardiovascular mortality** | 0.834 (0.785, 0.887) | 0.843 (0.791, 0.899) | 0.872 (0.816, 0.931) |
| Ln-CALLY |  |  |  |
| T1 | Ref | Ref | Ref |
| T2 | 0.797 (0.668, 0.951) | 0.728 (0.610, 0.869) | 0.799 (0.667, 0.957) |
| T3 | 0.621 (0.515, 0.747) | 0.626 (0.519, 0.755) | 0.693 (0.570, 0.844) |
| *P* for trend | <0.001 | <0.001 | <0.001 |

HR: hazard ratio

95% CI: 95% confidence interval

Model 1: no covariates were adjusted

Model 2: Adjusted for age, sex, and race

Model 3:Adjusted for age, sex, race, education, marital status, PIR, body mass index, smoking, drinking, moderate activity, vigorous activity, diabetes, hypertension, hyperlipidemia, cardiovascular disease, eGFR, ALT, AST and uric acid.
