# Supplementary material for: Negative association of C-reactive protein-albumin-lymphocyte index (CALLY index) with all-cause and cardiovascular mortality in population with CKD: the mediating role of biological age acceleration
Source: Ren Fail. 2025 Nov 18;47(1):2586892. doi: 10.1080/0886022X.2025.2586892 (PMC12632228; doi:10.1080/0886022X.2025.2586892)
Supplement: Supplementary Table 1.docx [file IRNF_A_2586892_SM5510.docx]

**Supplementary Table 1.** Association of Ln-CALLY with BioAgeAccel

|  | **β value** | **95% CI 95%** | ***P*-value** |
| --- | --- | --- | --- |
| Model 1 | -4.127 | (-4.561,-3.692) | <0.001 |
| Model 2 | -4.274 | (-4.699,-3.848) | <0.001 |
| Model 3 | -3.364 | (-3.750,-2.979) | <0.001 |

95% CI: 95% confidence interval

Model 1: no covariates were adjusted

Model 2: Adjusted for age, sex, and race

Model 3: Adjusted for age, sex, race, education, marital status, PIR, body mass index, smoking, drinking, moderate activity, vigorous activity, diabetes, hypertension, hyperlipidemia, cardiovascular disease, eGFR, ALT, AST and uric acid.
