# Supplementary material for: Negative association of C-reactive protein-albumin-lymphocyte index (CALLY index) with all-cause and cardiovascular mortality in population with CKD: the mediating role of biological age acceleration
Source: Ren Fail. 2025 Nov 18;47(1):2586892. doi: 10.1080/0886022X.2025.2586892 (PMC12632228; doi:10.1080/0886022X.2025.2586892)
Supplement: Figure legends.docx [file IRNF_A_2586892_SM5509.docx]

**Fig.1**

Flowchart of study participants. CALLY, C-reactive protein-albumin-lymphocyte.

Legend:NHANES, National Health and Nutritional Examination Surveys; CKD, chronic kidney disease.

**Fig. 2**

Kaplan-Meier survival curves for the effect of CALLY on long-term all-cause mortality (A) and cardiovascular disease mortality (B) in a CKD population.

**Fig. 3** Nonlinear associations of ln-CALLY with all-cause (A) and cardiovascular (B) mortality in CKD.

Legend:Adjusted for age, sex, race, education, marital status, PIR, body mass index, smoking, drinking, moderate activity, vigorous activity, diabetes, hypertension, hyperlipidemia, cardiovascular disease, eGFR, ALT, AST and uric acid.

**Fig 4.** Mediation analysis of the association between Ln-CALLY and mortality through BioAgeAccel.

Legend: (A) Mediation effect of BioAgeAccel on the association between Ln-CALLY and all-cause mortality. (B) Mediation effect of BioAgeAccel on the association between Ln-CALLY and cardiovascular mortality. All models were adjusted for age, sex, race, education, marital status, PIR, body mass index, smoking, drinking, moderate and vigorous physical activity, diabetes, hypertension, hyperlipidemia, cardiovascular disease, eGFR, ALT, AST, and uric acid.

**Fig 5.** Time-dependent ROC analysis comparing the predictive performance of CALLY, AIP, and SII for mortality in patients with CKD.

Legend: (A) Predictive ability for all-cause mortality at 5, 10, and 15 years. (B) Predictive ability for cardiovascular mortality at 5, 10, and 15 years. The area under the curve (AUC) with 95% confidence intervals is presented for each marker.
