# Supplementary material for: Negative association of C-reactive protein-albumin-lymphocyte index (CALLY index) with all-cause and cardiovascular mortality in population with CKD: the mediating role of biological age acceleration
Source: Ren Fail. 2025 Nov 18;47(1):2586892. doi: 10.1080/0886022X.2025.2586892 (PMC12632228; doi:10.1080/0886022X.2025.2586892)
Supplement: Supplementary Table 2.docx [file IRNF_A_2586892_SM5508.docx]

**Supplementary Table 2.** Association of BioAgeAccel with all-cause mortality and CVD mortality

|  | **HR** | **95% CI** | ***P***-value |
| --- | --- | --- | --- |
| **all-cause mortality** |  |  |  |
|  |  |  |  |
| Model 1 | 1.002 | (1.000, 1.005) | 0.024 |
| Model 2 | 1.012 | (1.010, 1.014) | <0.001 |
| Model 3 | 1.010 | (1.008, 1.012) | <0.001 |
|  |  |  |  |
| **CVD mortality** |  |  |  |
|  |  |  |  |
| Model 1 | 1.004 | (1.000, 1.008) | 0.046 |
| Model 2 | 1.014 | (1.010, 1.018) | <0.001 |
| Model 3 | 1.014 | (1.010, 1.018) | <0.001 |

95% CI: 95% confidence interval

Model 1: no covariates were adjusted

Model 2: Adjusted for age, sex, and race

Model 3: Adjusted for age, sex, race, education, marital status, PIR, body mass index, smoking, drinking, moderate activity, vigorous activity, diabetes, hypertension, hyperlipidemia, cardiovascular disease, eGFR, ALT, AST and uric acid.
